# Number of teeth and myocardial infarction and stroke among elderly never smokers

**DOI:** 10.1186/1477-5751-8-6

**Published:** 2009-04-22

**Authors:** Anna-Maija H Syrjälä, Pekka Ylöstalo, Sirpa Hartikainen, Raimo Sulkava, Matti L Knuuttila

**Affiliations:** 1 Department of Periodontology, Institute of Dentistry, University of Oulu, Oulu, Finland; 2 Oulu Health Centre, Oulu, Finland; 3 Department of Periodontology, Institute of Dentistry, University of Oulu, Oulu, Finland; 4 Faculty of Pharmacy, Kuopio Research Centre for Geriatric Care (Gerho), University of Kuopio, Kuopio, Finland; 5 Leppävirta Health Centre, Leppävirta, Finland; 6 School of Public Health and Clinical Nutrition. Division of Geriatrics, University of Kuopio, Kuopio, Finland; 7 Department of Periodontology, Institute of Dentistry, University of Oulu, Oulu, Finland; 8 Oral and Maxillofacial Department, Oulu University Hospital, Oulu, Finland

## Abstract

**Background:**

In most previous studies the association between number of teeth and cardiovascular diseases has been found to be stronger among younger age groups than in older age groups, which indicates that age may modify the association between number of teeth and cardiovascular diseases.

We investigated the association between tooth loss and atherosclerotic vascular diseases such as myocardial infarction and stroke in a homogeneous elderly population.

The study population was comprised of a subpopulation of 392 community-living elderly people who participated in the population-based Kuopio 75+ study. The data were collected through an interview, a structured clinical health examination and from patient records. The main outcome measures were a history of diagnosed myocardial infarction and diagnosed ischemic stroke. Prevalence proportion ratios (PPR) were estimated using generalised linear models.

**Results:**

Edentate subjects had a weakly, statistically non-significantly increased likelihood of a history of myocardial infarction and ischemic stroke compared with dentate subjects. Those with a large number of teeth had a slightly, but not statistically significantly increased likelihood of a history of myocardial infarction and ischemic stroke compared with those with a small number of teeth.

**Conclusion:**

These data did not show evidence that total or partial tooth loss would be associated with atherosclerotic vascular diseases such as myocardial infarction and ischemic stroke among an elderly population aged 75 years or older.

## Background

Cardiovascular diseases (CVD) are one of the main causes of death in the world, accounting for almost one third of all deaths world-wide [1] and about half of all deaths in Europe [2]. Cardiovascular diseases consist of heterogeneous groups of vascular diseases, with atherosclerotic vascular diseases being the commonest group. Although the risk factors of atherosclerotic vascular diseases include several risk factors such as abnormal lipids, hypertension, smoking and diabetes [3], a substantial proportion of cardiovascular events cannot be attributed to any of the risk factors [4].

During the past three decades, oral epidemiologists have been actively testing the hypothesis that oral infections may be aetiological factors in atherosclerotic vascular diseases. Different explanatory variables such as periodontal pocket depth, clinical attachment loss or different indices have been used to measure the extent and/or severity of oral infection. Tooth loss, measured by number of teeth, has also been used as an explanatory variable, especially in situations where no other form of data is available.

In earlier studies, tooth loss has been associated with atherosclerotic diseases such as myocardial infarction (MI) [5,6], coronary heart disease [7,8] and stroke [9,10]. In addition, total tooth loss, edentulism, has been associated with fatal coronary heart disease [11], stroke/transient ischemic attack [12], stroke [13] and nonhemorrhagic stroke as well as all cerebrovascular accidents [14]. On the other hand, other studies have failed to show any association between number of teeth and myocardial infarction [15,16], myocardial infarction or angina pectoris or unstable angina [17] or coronary heart disease [18]. The association between tooth loss and cardiovascular diseases has been suggested to be due to different reasons, such as previous periodontal infections, caries, bacteraemia related to extractions, change in diet followed by tooth loss and biases such as selection bias, information bias and confounding related to attitudinal, behavioural and biological factors in common [19].

Most previous studies involve heterogeneous study populations that include both middle-aged and elderly persons; typically the mean age is under 70 years. Studies that have investigated the association in different age groups have shown that the association between number of teeth and cardiovascular diseases is stronger among younger age groups than in older age groups [9,20], which indicates that age may modify the association between number of teeth and cardiovascular diseases. Our aim was to study whether there is an association between number of teeth and atherosclerotic diseases such as myocardial infarction and ischemic stroke among a homogenous study population aged 75 years or older.

## Results

The descriptive statistics of the study population are shown in Tables 1 and 2.

**Table 1:** Sociodemographic characteristics of the participants

Variable	All (n = 390) %	Dentate (n = 169) %	Edentate (n = 221) %
**Gender**			
Proportion of males	13.1	19.5	8.1
**Marital status**			
Married	22.1	29.0	16.7
Widowed	59.5	50.3	66.5
Living alone	66.9	63.3	69.7
**High education**	19.2	31.4	10.0

**Table 2:** Cardiovascular diseases and potential risk factors among dentate and edentate participants

Variable	All (n = 390)	Dentate (n = 169)	Edentate (n = 221)
**Cardiovascular diseases**			
Myocardial infarction (%)	29.5	27.8	30.8
Stroke (%)	8.0	7.1	8.6
**Potential risk factors of cardiovascular diseases**			
BMI: mean (SD)	26.4 (4.6)	25.9 (4.5)	26.9 (4.6)
BMI: ≥ 25 kg/m^2^* (%)	59.5	55.6	62.4
Serum total cholesterol: mean (SD)	5.7 (1.2)	5.7 (1.2)	5.8 (1.2)
Serum total cholesterol: ≥ 5.2 mmol/l* (%)	65.1	63.3	66.5
Serum HDL cholesterol: mean (SD)	1.5 (0.4)	1.5 (0.4)	1.5 (0.4)
Serum HDL cholesterol: ≤ 1.04 mmol/l* (%)	10.8	8.3	12.7
Serum triglycerides: mean (SD)	1.5 (0.8)	1.4 (0.8)	1.6 (0.8)
Serum triglycerides: ≥ 2.26 mmol/l* (%)	13.1	10.7	14.9
Diabetes (%)	17.4	11.8	21.7
Blood glucose: mean (SD)	5.6 (1.4)	5.5 (1.2)	5.8 (1.5)
Hypertension (%)	50.3	51.5	49.3
Alcohol consumption: Proportion of those who drink alcohol (%)	37.0	39.6	35.0
Physical activity:			
No exercise (%)	45.1	41.7	47.7
Slow walking or intense exercise 2–3 times a week (%)	55.0	58.3	52.3

*Serum triglycerides ≥ 2.26 mmol/l, serum HDL cholesterol ≤ 1.04 mmol/l, serum total cholesterol ≥ 5.2 mmol/l, and BMI ≥ 25 kg/m^2 ^are regarded as CVD risk factors [29,30].

After controlling for gender, age, basic education, diabetes, hypertension, smoking, alcohol consumption, physical activity, body mass index (BMI), serum triglycerides and serum high-density lipoprotein (HDL) cholesterol, dentate persons have a slightly, non-significantly decreased likelihood of having a history of myocardial infarction (PPR 0.9 95% CI: 0.5–1.8) or ischemic stroke (PPR 0.9 95% CI: 0.2–2.8) when compared with edentulous subjects. Number of teeth was weakly and statistically non-significantly associated with a history of myocardial infarction (PPR 1.01 95% CI: 0.97–1.05) and ischemic stroke (PPR 1.02 95% CI: 0.94–1.08) (Table 3).

**Table 3:** Association between number of teeth and presence of cardiovascular diseases among never-smokers.

Variable	Myocardial infarction	Myocardial infarction	Stroke	Stroke
	Crude PPR (CI)	PPR (CI) *	Crude PPR (CI)	PPR (CI) *
Number of teeth (continuous)	1.00 (0.98–1.02)	1.01 (0.97–1.05)	0.99 (0.95–1.03)	1.02 (0.94–1.08)
Dentate *vs. *edentate	0.9 (0.6–1.3)	0.9 (0.5–1.8)	0.8 (0.4–1.7)	0.9 (0.2–2.8)
Gender (male *vs. *female)	1.5 (0.9–2.3)	1.8 (1.0–3.1)	1.0 (0.3–2.5)	0.8 (0.2–2.0)
Age	1.06 (1.02–1.10)	1.07 (1.02–1.11)	1.00 (0.92–1.08)	0.97 (0.87–1.06)
Education (low *vs. *high)	1.1 (0.7–1.9)	1.4 (0.8–2.5)	2.2 (0.8–9.2)	2.7 (0.8–11.1)
Diabetes (no *vs. *yes)	0.6 (0.4–0.9)	0.5 (0.3–0.9)	0.4 (0.2–1.0)	0.5 (0.2–1.1)
Hypertension (no *vs. *yes)	0.9 (0.6–1.3)	1.0 (0.7–1.5)	0.6 (0.3–1.3)	1.0 (0.4–2.1)
BMI	1.04 (1.00–1.08)	1.03 (0.99–1.08)	1.00 (0.93–1.08)	0.97 (0.89–1.05)
Serum HDL cholesterol	0.83 (0.5–1.3)	1.50 (0.84–2.60)	0.32 (0.11–0.81)	0.23 (0.06–0.78)
Serum triglycerides	1.23(1.00–1.46)	1.13 (0.90–1.43)	1.23 (0.82–1.69)	0.89 (0.48–1.34)
Alcohol consumption (no *vs. *yes)	1.3 (0.9–1.9)	1.3 (0.8–2.0)	0.8(0.4–1.7)	0.8 (0.4–1.7)
Physical activity (no *vs. *yes)	1.3 (0.9–1.9)	1.1 (0.7–1.7)	1.3 (0.6–2.6)	1.2 (0.5–2.3)

Unadjusted and adjusted prevalence proportion ratios (PPR) and 95% confidence intervals (CI).

*Adjusted for gender, age (as a continuous variable), basic education, diabetes, hypertension, BMI (as a continuous variable), serum HDL cholesterol (as a continuous variable), serum triglycerides (as a continuous variable), alcohol consumption, physical activity.

**Figure 1:**
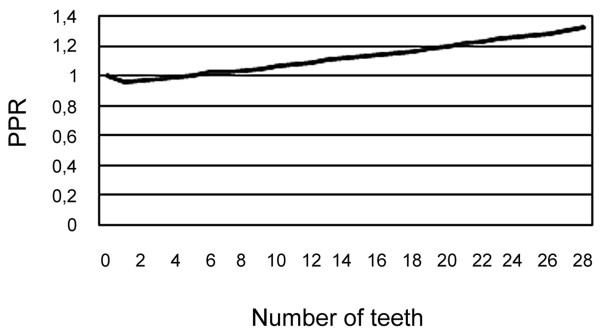
**Prevalence proportion ratios (PPR) of myocardial infarction in relation to the number of teeth**.

**Figure 2:**
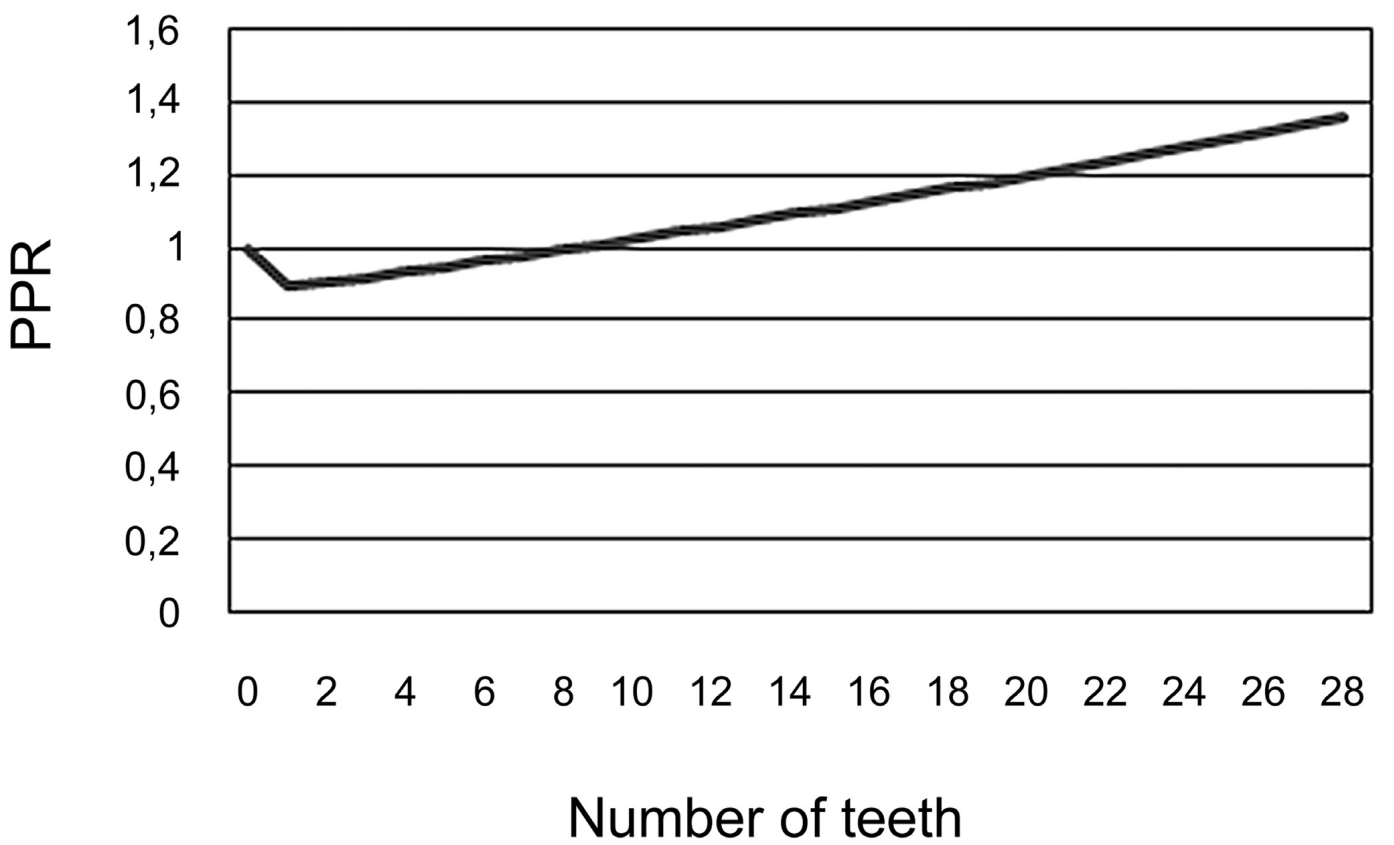
**Prevalence proportion ratios (PPR) of stroke in relation to the number of teeth**.

The joint effect of dentulous/edentulous and number of teeth is presented in figures 1 and 2. It can be seen that the joint effect of these variables forms a J-shaped curve. From the figures it can be seen that the likelihood of a history of both MI and stroke is lowest among those with a small number of teeth. The equivalent point when compared with edentulous subjects was 5 teeth for MI and 8 teeth for stroke.

Due to unequal gender distribution, interactions between gender and explanatory variables were tested. The p-values for interaction terms between gender and number of teeth was 0.89 and between gender and dentate *vs*. edentate, 0.87, for myocardial infarction. For stroke, the corresponding values were 0.26 and 0.57.

## Discussion

The results did not provide evidence that partial tooth loss or edentulousness would be associated with myocardial infarction or stroke. In fact, these data showed that those with a large number of teeth might be more likely to have a history of diagnosed myocardial infarction and stroke, and that those with small residual dentition, *i.e. *subjects who had lost most of their teeth, might be least likely to have a history of MI and stroke. However, it must be emphasised that such extrapolations of the estimates of multivariate models as seen in figures 1 and 2 are as uncertain as the original estimates.

In order to reduce the effect of confounding, we adjusted for age, several classical cardiovascular risk factors, educational level and behavioural factors, which all were unequally distributed between dentate and edentate. In addition, we restricted the study to subjects who had never smoked, since complete control of smoking is otherwise considered impossible. The study population was quite homogeneous with regard to ethnic origin, age (75 or over), geographical distribution (residents in the town of Kuopio) and physical capacity (home-dwellers, not institutionalised), which also reduced confounding. However, despite profound adjustment and restrictions, the possibility that some residual confounding existed cannot beexcluded, especially related to those factors that are difficult or impossible to fully conceptualise, such as behavioural, attitudinal and socioeconomic factors. An additional reason why we did not find any significant associations could be that residual confounding related to strong determinants, such as education, for example, masks the effect of less powerful determinants.

The participation rate of this study was high, 86%, which reduced the possibility that participation in the study would be responsible for the findings. There was some bias related to the participation of subjects; the elderly people who did not participate in the study were somewhat older than the participants. Regarding gender, there was no essential difference in gender distribution between the participants and non-participants. The participants of this study were home-dwelling but not institutionalised subjects, meaning that the participants represented a selected subgroup of this age cohort in terms of general health status. This in turn may lead to a situation where high-risk subjects may not have been included in the study, because severe health problems may lead to institutionalisation, hospitalisation or premature deaths. Such selective survival leads to survival bias, which possibly prevents detection of a true association between tooth loss and atherosclerotic vascular diseases. Bias related to survival emphasises the need to perform incidence-type cohort studies instead of cross-sectional studies.

In this study, information was collected from patient records, which reduces the possibility that the findings were caused by an imbalance in information.

### Limitations of the study

This study is a secondary analysis of data collected for other purposes. This has several implications. Firstly, we had a limited number of subjects; the original sample numbered 700, of which a fairly high proportion (86%) participated in the study. After restriction to never smokers, only 392 remained, and it is possible that this was too small a number to show any statistically significant differences in our estimates (dentate *vs*. edentate, number of teeth as a continuous variable). However, it must be remembered that estimates themselves are not dependent on the size of the study. Secondly, the fact that this was a secondary analysis also meant that oral examination was by no means comprehensive, and the number of remaining teeth, *i.e*. tooth loss, was the only suitable explanatory variable. This variable has a certain well-known shortcoming, namely, it is an indirect, unspecific and unreliable measure of a history of oral diseases. Previous studies suggest that periodontitis is common among elderly people [21] and they have often lost their teeth due to periodontal infection [22,23]. However, it must be noted that we did not have any information about the reasons why teeth were lost, and tooth loss is most likely a confounded measure of oral health, since teeth probably have also been lost for several reasons, such as treatment of larger fractures, large caries lesions, acute pain or abscesses due to pulpal or periodontal involvement, for example. Moreover, a negative attitude towards preservation of natural teeth as well as economic constraints might lead to situations where subjects with low socioeconomic status prefer tooth extraction instead of more conservative treatment.

A self-evident limitation of this study is the use of cross-sectional data, which means the temporal sequence between explanatory and outcome variables cannot be determined. Myocardial infarction and stroke are end points in disease processes that have started years earlier. Possible causes of tooth loss, dental caries and periodontitis, for instance, are chronic diseases that may last for years or decades and may gradually lead to tooth loss. These aspects make it difficult to determine temporal sequence. On the other hand, temporal sequence can be in the opposite direction from what is expected. For example, severe heart disease may decrease capability to perform daily oral self-care routines. This in turn increases the incidence of dental caries and periodontal disease, which among severely diseased elderly people easily leads to extractions.

It must be emphasised that these data consisted of mostly women, which based on their higher life expectancy, is not surprising. Gender distribution also did not correspond to the total Finnish population in that age group, which most likely is due to the fact that smoking in those age groups has been commoner among men than women. On the other hand, it must be pointed out from the point of view of generalisation that we did not find any essential interaction between explanatory variables and gender, which suggests that the results can be generalised to persons of both genders aged 75 years or more who had never smoked.

Lastly, an obvious weakness of the study is that the clinical health examinations, which included a partial oral examination, were done by a geriatrician, and intra-examiner reliability was not evaluated. The same geriatrician did all the examinations, which eliminates variation due to disagreement between examiners, which is often present in large field studies. The fact that the same geriatrician did the clinical health examination meant that the geriatrician may have been aware of the disease history of the subject when performing the oral examination. Whether this had any affect on the results is not known, but the fact that the aim of this paper did not originally belong to the aims of this study project, means that bias in registration is therefore difficult to imagine. From the point of view of validity, it must be emphasised that the exposure variable, *i.e*. number of teeth, is quite simple and can be assessed fairly reliably even by non-dentists. According to previous studies, simple detectable dental conditions, also when self-reported, correspond quite well with clinical findings [24,25].

## Conclusion

These data suggest that in an elderly population, partial tooth loss or edentulousness is not associated with a history of myocardial infarction and ischemic stroke. The fact that we did not find such associations could be interpreted that there is no causal association among the elderly, or that association among the elderly is too weak to be observable in the presence of other determinants, or that biases such as different forms of selection bias or confounding may have prevented us from detecting the association between tooth loss and a history of myocardial infarction and stroke.

## Materials and methods

### Study population

The study population consisted of community-living participants in the population-based Kuopio 75+ study, which focused on the prevalence of chronic diseases, use of medication and functional capacity among persons aged 75 years or more. The study population was a random sample from a census register consisting of 700 persons drawn from the total population (n = 4518) born before 1 January 1923 and living in Kuopio on 1 January, 1998.

In 1998 a geriatrician and a trained nurse conducted structured clinical examinations and interviews among 601 persons (86% of the random sample). Of the sample, 79 subjects refused, 5 were not contacted, and 15 were dead. Elderly persons living in long-term facilities (n = 78) were excluded. The study design has been described in more detail in earlier reports [26,27]. In this paper, we restricted the study population to subjects who had never smoked, and after that restriction the study population consisted of 392 home-dwelling persons aged 75 or more.

Written informed consent for the study was obtained from the subjects or their relatives. The ethics committee of the Kuopio University Hospital approved the study protocol.

### Interview

Data on sociodemographic variables, physical health and health behaviour were collected using structured interviews. Education was categorised according to the subjects' basic education (classification for analysis: low education; elementary school or lower, some other form of education *vs. *high education; junior secondary school, senior secondary school, matriculation). Health behaviour variables included alcohol consumption (classification for analysis: alcohol consumption *vs. *no alcohol) and physical activity (classification for analysis: no exercise *vs. *slow walking or intense exercise 2–3 times a week).

### Clinical health examination

The clinical health examination was done by a geriatrician. Myocardial infarction and stroke were determined from patient records. Previous myocardial infarction was determined on the basis of a recorded medical diagnosis or changes in an electrocardiogram suggestive of infarction. Stroke was determined based on verified occurrence of an ischemic stroke as a type of first cerebral palsy. Diabetes was determined on the basis of a recorded medical diagnosis or reimbursable medication. Hypertension was determined on the basis of a recorded medical diagnosis. Body mass index (BMI) was determined using body weight and height (body weight in kilograms/height in meters^2^), which were measured during the clinical examination. Basic laboratory tests were performed and analysed using standard enzymatic methods.

The clinical health examination included a partial oral examination, which was done by a geriatrician under the guidance of a dentist. It included recording the presence of a removable denture, the number of teeth, carious teeth and dental radix, mucosal lesions and a saliva sample.

### Statistical analyses

All analyses were performed using the SAS Genmod procedure. Adjusted prevalence proportion ratios (PPR) were estimated using Poisson distribution, a log link function, a REPEATED statement and an unstructured correlation matrix [28-30]. In addition, risk estimates were calculated for different numbers of teeth as a joint function of two variables (dentulous/edentulous) and number of teeth (continuous). This was performed using an ESTIMATE statement in the SAS Genmod procedure.

Potential confounders included generally accepted risk factors of CVD or risk indicators of CVD that were available and were unequally distributed. The following variables were chosen: gender, age, basic education, diabetes, hypertension, alcohol consumption, physical activity, BMI, serum triglycerides and serum HDL cholesterol. Interactions between gender and the main explanatory variables (dentulous/edentulous) and number of teeth were tested.

## Competing interests

The authors declare that they have no competing interests.

## Authors' contributions

A-MS mainly did the drafting. She participated in the interpretation of the data. She revised the article and participated in the final approval of the article. PY did the statistical analysis, also did drafting and revision and participated in the final approval. SH contacted A-MS and gave the data to her for analysis. She revised the article and participated in the final approval. RS planned the study and participated in the implementation of the study. He revised the article and participated in the final approval. MK participated in the interpretation, revision and final approval of the article.
